# Effect of Early Life Stress on the Epigenetic Profiles in Depression

**DOI:** 10.3389/fcell.2020.00867

**Published:** 2020-10-07

**Authors:** Ming Li, Xiying Fu, Wei Xie, Wanxu Guo, Bingjin Li, Ranji Cui, Wei Yang

**Affiliations:** Jilin Provincial Key Laboratory on Molecular and Chemical Genetic, Second Hospital of Jilin University, Changchun, China

**Keywords:** early life stress, epigenetic, depression, DNA methylation, HPA axis

## Abstract

Depression is one of the most common mental disorders and has caused an overwhelming burden on world health. Abundant studies have suggested that early life stress may grant depressive-like phenotypes in adults. Childhood adversities that occurred in the developmental period amplified stress events in adulthood. Epigenetic-environment interaction helps to explain the role of early life stress on adulthood depression. Early life stress shaped the epigenetic profiles of the HPA axis, monoamine, and neuropeptides. In the context of early adversities increasing the risk of depression, early life stress decreased the activity of the glucocorticoid receptors, halted the circulation and production of serotonin, and reduced the molecules involved in modulating the neurogenesis and neuroplasticity. Generally, DNA methylation, histone modifications, and the regulation of non-coding RNAs programmed the epigenetic profiles to react to early life stress. However, genetic precondition, subtypes of early life stress, the timing of epigenetic status evaluated, demographic characteristics in humans, and strain traits in animals favored epigenetic outcomes. More research is needed to investigate the direct evidence for how early life stress-induced epigenetic changes contribute to the vulnerability of depression.

## Introduction

Depression is one of the most common mental health problems worldwide. According to a report from the Global Burden Data, the mental disorder was on the list of the leading causes of death (GBD 2017 Causes of Death Collaborators, 2018). Hitherto, though depression is characterized by a disturbance of the hypothalamic-pituitary-adrenal (HPA) axis, monoamine or neuroplasticity, and neurogenesis, how these biological events react to environment changes has not been fully illustrated (Malhi and Mann, 2018). Epigenetics is the study of heritable and genetic events added to traditional genetics. Epigenetics aims to explore the alterations of DNA methylation (DNAm), histone modification, and non-coding RNAs. In other words, epigenetic changes do not alter the original DNA sequence and it perfectly explains the interaction between the environment and genetics (Dupont et al., 2009). Early life stress (ELS) is a prevalent experience that happens before birth or in early postnatal life. On the one hand, ELS increased the risk of depression or worsened depressive symptoms. On the other hand, ELS caused epigenetic changes in the HPA axis, serotonin, dopamine, and some neuropeptides (McGowan et al., 2009; Roth et al., 2009; Bai et al., 2012; Kang et al., 2013; Ouellet-Morin et al., 2013; Zhang et al., 2015; Bahi, 2016; Bustamante et al., 2016; Tyrka et al., 2016; Williams et al., 2016; Wang et al., 2017). Therefore, epigenetic events bridge the connection between ELS and depression and some studies have proven the effect of epigenetic changes on the accumulating risk of developing depression in those who were subjected to ELS (Kang et al., 2013; Melas et al., 2013; Wankerl et al., 2014; Cecil et al., 2016; Tyrka et al., 2016; Dickson et al., 2018; Opel et al., 2019). However, though being exposed to ELS increased depressive incidents, not all individuals who suffered ELS acquired depression. Genetic precondition determines whether exhibited pro-depressive epigenetic alteration or stress-resistant profiles confronted ELS. Moreover, subtypes of ELS, gender, exposed timing, and duration matter in the role of ELS in depression (Cecil et al., 2016; McCoy et al., 2016; Frodl et al., 2017; Reus et al., 2017). In this system review, we will discuss the effect of ELS on epigenetic alterations, which mainly facilitated the occurrence and development of depression in two independent sections, and in each section, we will discuss DNAm, histone modification, and RNA transcripts.

## Overview of Epigenetics

Epigenetic events mainly refer to the alterations of DNAm, histone modification, and microRNA (miRNA) (Dupont et al., 2009). Among them, DNAm is the most well-defined and thoroughly investigated (Ouellet-Morin et al., 2013; Montirosso et al., 2016; Tyrka et al., 2016; Janusek et al., 2017; Wikenius et al., 2019). DNAm mostly occurs in CpG sites. Generally, most cytosine–phosphate–guanine (CpG) islands (CG enriched) remained hypomethylated but the other CpG sequences remained hypermethylated. Methylation or demethylation potentially suppresses or promotes gene transcription, respectively (McGowan et al., 2009; Melas et al., 2013; Ouellet-Morin et al., 2013; Peng et al., 2018). Secondly, histone modification includes acetylation, methylation, ubiquitylation, phosphorylation, SUMOylating, ribosylation, and citrullination. Among them, acetylation is the most highly studied in mental disorders (Levine et al., 2012; Seo et al., 2016; Wang et al., 2018; Karen and Rajan, 2019). Histone modification can loosen or tighten the chromosome to alter transcription. miRNAs can directly or indirectly regulate genes and miRNAs were also upregulated or downregulated in depressed patients who had ELS (Dupont et al., 2009).

## Definition of Depression

Depression is a prevalent mental illness and it leads to an increased health burden worldwide (GBD 2017 Causes of Death Collaborators, 2018). In a hypothesis that described the progression of depression, the HPA axis was most prominent. The HPA axis plays a critical role in the response to stress, and a disturbance of the HPA axis results in vulnerability for depression (Bjorkenstam et al., 2017; Reus et al., 2017). It is well-documented that ELS enhanced the vulnerability for depression (Kim et al., 2013; Culpin et al., 2015; St Clair et al., 2015; Airagnes et al., 2016; Williams et al., 2016; Bjorkenstam et al., 2017; Dahl et al., 2017; Opel et al., 2019; Tracy et al., 2019). Depressed subjects may bear the increased level of plasma cortisol of excessive stress-induced and impaired functions of the glucocorticoid receptors (GR) (Malhi and Mann, 2018). Excessive cortisol and impaired GRs increased the susceptible response to stress. However, some depressed patients did not obtain cortisol changes and therefore other biomedical alternations may also account for the occurrence of depression. Serotonin or 5-hydroxytryptamine (5-HT), noradrenaline, and dopamine are monoamines involved in the progression of depression. Selective serotonin reuptake inhibitors regulate the synaptic plasticity, improving depressive symptoms in depressed patients (Kinnally et al., 2011; Kang et al., 2013; Ouellet-Morin et al., 2013). Besides, some neuropeptide-modulated neurogenesis and neuroplasticity. Their alterations increased the risk for depression and worsened the response to antidepressants, of which, the brain-derived neurotrophic factor (BDNF) was heatedly investigated (Roth et al., 2009; Bai et al., 2012; Doherty et al., 2016). Besides the hypothesis, pro-inflammation was presumed to deteriorate or even accelerate the progression of depression (Janusek et al., 2017). Pro-inflammatory status was found among the depressed and possibly the pro-inflammation interacted with the dysfunctional HPA axis (Malhi and Mann, 2018). Regarding the mechanism of depression, genetic factors cannot be ignored because of the disparity among populations and sex in dealing with stress (Perroud et al., 2011). Epigenetic profiles are the internal temperament that defines external personal characteristics. Epigenetic changes may be long-lasting (Dickson et al., 2018; Roberts et al., 2018). ELS happens in a prenatal or early postnatal time frame but is attributed to the personal susceptibility to depression (St Clair et al., 2015; Bjorkenstam et al., 2017; Reus et al., 2017). Therefore, ELS is the perfect example to better explain the epigenetic role in the evolution of depression.

## Epigenetic Studies Focused on Human Beings

Robust clinical studies suggested that ELS increased the risk of depression. Epigenetic modification underlies the regulation of the HPA axis, monoamine, and neuropeptides. The epigenetic studies were either conducted in a site-specific or genome-wide pattern. Of those studies, DNAm was richly demonstrated. Firstly, we introduce epigenetic regulation focused on interesting regions. McGowan and his colleagues found that the neuron-specific glucocorticoid receptor (NR3C1) promoter was hypermethylated in the postmortem hippocampus of suicide victims with a history of childhood abuse compared to those victims without child abuse (McGowan et al., 2009) and increased DNAm of the exon 1F NR3C1 promoter was also observed in the peripheral blood of patients with major depressive disorders (MDD) who suffered ELS (Perroud et al., 2011). The patch hypermethylation within the NR3C1 promoter resulted in the decreased expression of NR3C1 (McGowan et al., 2009; Perroud et al., 2011). Furthermore, the severity and the number of types of ELS positively correlated with the exon 1F NR3C1 DNAm in MDD adults (Perroud et al., 2011). Except for childhood adversities, prenatal distress also modulated the DNAm of the exon 1F NR3C1 promoter of newborns. Prenatal depressive symptoms increased the exon 1F NR3C1 DNAm in male infants rather than in female infants (Braithwaite et al., 2015). Likewise, the composite measurement of child maltreatment was positively correlated with the DNA methylation of exons 1D and 1F in the promoter of the GR gene in preschool children (Tyrka et al., 2015). Moreover, compared with suicides without childhood abuse, the exon 1B and 1C, but not 1H GR promoters were also hypermethylated while the expression of the variants 1B and 1C were decreased in the hippocampus of suicides with ELS exposure (Labonte et al., 2012). Additionally, in a study of monozygotic twin pairs, Palma-Gudiel et al. (2018) demonstrated that increased exon 1D NR3C1 CpG-specific methylation was related to depressive symptoms and decreased hippocampal connectivity. Moreover, another monozygotic twin study revealed that DNAm at the NR3C1 mediated the association between childhood trauma and depression (Peng et al., 2018). In short, the publications we mentioned above supported that fact that an increased methylated GR gene or regions in individuals with ELS and the level of DNAm positively correlated with the severity of ELS or specific subgroups of ELS. The hypermethylation of GR genes likely dysregulated the HPA axis and made the subject more vulnerable to stress and possibly more likely to develop depression. In contrast to this opinion, Tyrka et al. (2016) proposed that reduced DNAm of the NR3C1 was associated with childhood maltreatment and depressive disorders in adults. In this study, ELS and current or post depressive or anxiety disorders were correlated with a reduction in exon 1F NR3C1 promoter methylation either at individual CpG sites or across the gene. Moreover, the altered NR3C1 DNAm induced a blunt cortisol response to stress (Tyrka et al., 2016). Likewise, Alexander et al. (2018) found an increased DNAm of the NR3C1 exon 1F promoter in healthy adults with childhood trauma exposure. However, this change seemed independent of the cortisol response to stress in those unexposed and mildly or moderately ELS-exposed individuals (Alexander et al., 2018). What is more, one study that enrolled healthy women aged 40 + assumed no significant associations between childhood adversity and DNAm in the NR3C1 promoter but higher DNAm in the ERα shore correlated with higher levels of adversity (Fiacco et al., 2019). In addition, childhood maltreatments and MDD induced different epigenetic changes at the NR3C1 promoter. Specifically, childhood maltreatments were associated with increased DNAm in an EGR1 transcription factor binding site (NGFI-A), whereas MDD was associated with a decrease in DNA downstream of NGFI-A. It was only childhood maltreatments, rather than the depressive symptoms, that were associated with reduced NR3C1 expression (Bustamante et al., 2016). Therefore, though most studies supported that ELS increased the NR3C1 DNAm and possibly that the epigenetic alterations resulted in a blunt HPA axis, emerging evidence indicated that even ELS altered NR3C1 DNAm, this molecular change may not be powerful enough to develop depression (Tyrka et al., 2016; Alexander et al., 2018; Fiacco et al., 2019). It was reported that depressive patients harbored different profiles of the HPA axis, which might be due to a feature of the NR3C1 DNAm (Malhi and Mann, 2018). Furthermore, demographic characteristics (i.e., age and gender), types and severity of ELS (physical abuse, sexual abuse, emotional abuse, physical and emotional neglects), tissue specificity (i.e., subregions of the brain, blood), dynamic changes in epigenetic profiles (interacting with continuous environment actions), and study design contributed to this discrepancy (McGowan et al., 2009; Perroud et al., 2011; Kember et al., 2012; Bustamante et al., 2016; Cecil et al., 2016; Tyrka et al., 2016; Alexander et al., 2018; Palma-Gudiel et al., 2018; Peng et al., 2018; Fiacco et al., 2019). Besides, because research focusing on the figure of histone modification and non-coding RNA within NR3C1 is lacking, it is unknown whether DNAm, histone modification, and non-coding RNA coordinate or disintegrate with each other. Therefore, ELS probably altered the epigenetic profile of the NR3C1, but whether this change mediated the association between ELS and depression is in debate. ELS reshaped the epigenetic figures of the HPA axis, especially NR3C1, and mediated the cortisol response to the stress (McGowan et al., 2009; Perroud et al., 2011; Tyrka et al., 2016; Palma-Gudiel et al., 2018). Increased NR3C1 DNAm was assumed to downplay the function of GRs and exert a blunt cortisol level in response to stress (Palma-Gudiel et al., 2018; Peng et al., 2018).

Further, except for the evidence focused on the epigenetic alterations of the GR genes, the genes involved in the regulation of the HPA axis were also explored. FKBP5 (FK506 binding protein 5), the functional glucocorticoid response element, encodes the protein that interacts with corticoid receptors. Elevated glucocorticoids activate the expression of FKBP5 or inversely, the elevated FKBP5 reduces the activity of GRs. Klengel et al. (2013) proposed that allele-specific FKBP5 DNA demethylation mediated gene–childhood trauma interactions. The demethylation event resulted in long-term dysregulation of the stress response and therefore increased the risk of developing stress-related psychiatric disorders in adulthood (Klengel et al., 2013; Tozzi et al., 2018). Moreover, according to a study of epigenome-wide blood DNAm, the DNAm of the kit ligand gene (KITLG) strongly medicated the relationship between childhood trauma and cortisol stress reactivity in humans (Houtepen et al., 2016). Therefore, with regard to the clinical investigation, except for the evidence of directly modified GRs, changes in the genes that interacted with GRs were also observed. It is possible that the genetic background, i.e., polymorphism, modulated the function of the GRs and their binding or responding elements when individuals were exposed to ELS and the subsequent risk of depression (Klengel et al., 2013; Houtepen et al., 2016; Peng et al., 2018; Tozzi et al., 2018).

Regarding the epigenetic changes of the 5-HT genes, SLC6A4 (solute carrier family 6 member 4) encodes the protein that transports the neurotransmitter serotonin from synaptic spaces into presynaptic neurons. MAO-A (monoamine oxidase A) encodes the enzymes that catalyze the monoamine, such as dopamine, norepinephrine, and serotonin. In other words, MAO-A was designed to eliminate monoamine. Therefore, SLC6A4 terminates the synaptic actions of serotonin. Current evidence supported that ELS was correlated with increased SLC6A4 DNAm and hence reduced the level of SLC6A4 (Kang et al., 2013; Ouellet-Morin et al., 2013; Wankerl et al., 2014). It is likely though that decreased SLC6A4 briefly accumulated the 5-HT in the synaptic cleft, the activated MAO-A enhanced the clearance in the absence of 5-HT recycle (Melas et al., 2013). Therefore, it is the increased SLC6A4 DNAm and decreased MAO-A that synergistically lessens the normal function of 5-HT. Therefore, weak 5-HT activity can be associated with depressive symptoms. Wang et al. suggested that childhood physical aggregation increased SLC6A4 DNAm in peripheral white blood cells and hypermethylated CpG sites reflecting serotonin synthesis in human brains (Wang et al., 2012). Kang and colleagues addressed that the hypermethylation of the SLC6A4 promoter was associated with childhood adversities and worse clinical presentations of 108 depressive patients. However, the methylation status was not correlated with treatment outcomes (Kang et al., 2013). Moreover, a longitudinal study of discordant monozygotic twins demonstrated that increased SLC6A4 DNAm was associated with bullying victimization. Children with a higher SLC6A4 DNAm level had impaired glucocorticoid-medicated feedback and it was assumed that this was linked to depression (Ouellet-Morin et al., 2013). Moreover, this study revealed that even patients free of bullying events, increased methylation level was observed at the age of 5 years, while this increasing trend vanished at the age of 10 in non-bullied patients. Whereas in the group of bullying victimization, higher SLC6A4 DNAm was still noticed at the age of 10 compared with the non-bullied monozygotic twins across the same time frame (Ouellet-Morin et al., 2013). Furthermore, compared with full-term infants, greater socio-emotional stress was associated with increased SLC6A4 promoter region methylation in 3-month-old very preterm infants (Montirosso et al., 2016). Moreover, very preterm infants were exposed to more stress in the very early stages of life and the subsequent alteration of SLC6A4 DNAm correlated with a reduced anterior temporal lobe (Fumagalli et al., 2018). Concerning female adults who harbored the allele MAOA-L and were exposed to childhood adversities concurrently, depression was more likely to develop. These females obtained decreased MAO-A DNAm and epigenetic modification was negatively correlated with childhood adversities (Melas et al., 2013). However, Wankerl et al. illustrated that maternal prenatal stress and child maltreatment were associated with reduced SLC6A4 mRNA expression, but the declination of SLC6A4 mRNA level was not likely mediated by the DNAm of the CpG island of the gene in healthy young adults (Wankerl et al., 2014). Therefore, though healthy individuals were subjected to early tragedies, the epigenetic mediation in these healthy persons helped to reduce the risk of developing depression.

Apart from the HPA axis and serotonin system, epigenetic regulation also presented at inflammation sites and in some neuropeptides. Depression often presents pro-inflammation and decreased neurogenesis. As for the pro-inflammatory profile, it was reported that elevated pro-inflammatory factors were associated with childhood adversities. Janusek et al. (2017) reported that the reduced DNAm of the IL-6 promoter was associated with childhood trauma in adult African American men. The reduced IL-6 promoter was accompanied by an increased acute stress-induced IL-6 response, and a blunted cortisol response. On the other hand, ELS seemed to contribute to the increased DNAm of BNDF in individuals with ELS, and this epigenetic alteration was long-lasting and possibly impacted the offspring of the females (Braithwaite et al., 2015; Peng et al., 2018). Furthermore, the functional single nucleotide polymorphisms (SNPs) of BDNF were moderated by the DNAm values at BDNF specific promoter sites and the interaction between SNPs and DNAm enhanced the susceptibility to depression (Ferrer et al., 2019). Therefore, current research suggested that ELS could alter the epigenetic profiles to present pro-inflammatory functions and reduce neurogenesis. However, more investigations should be conducted to prove that epigenetic alterations are associated with the risk of depression.

Apart from the research that were directly linked to the previously mentioned site-specific epigenetic changes, genome-wide studies were also performed. It was reported that in a group of French-Canadian men with severe childhood abuse, 362 promoters were differently methylated, and some involved in the neural plasticity (Labonté et al., 2012). Likewise, Suderman et al. (2014) revealed that 997 gene promoters were differentially methylated in adult men exposed to childhood abuse. Of those regulated genes, some involved in the key cell pathways were associated with transcriptional regulation and development. Moreover, ELS was associated with different methylated values at 2868 CpG sites in maltreated children. However, genes were involved in the epigenetic changes linked largely to non-mental disease (Yang et al., 2013). Contrasting with the epigenetically altered genes in maltreated children, maltreated young adults obtained several stress-related epigenetic genes. Besides, Cecil et al. demonstrated that different subtypes of ELS correlated with various epigenetic characters (Cecil et al., 2016). As for the possibility of maternal mental health affecting the epigenetic figure of their offspring, Roberts et al. (2018) suggested that exposure to childhood abuse was associated with human sperm DNAm. However, a longitudinal genome-wide study suggested no significant genome-wide association between maternal depressive symptoms and infant DNAm (Wikenius et al., 2019). Therefore, with regard to the genome-wide epigenetic alteration, variable profiles were more noticeable. Possibly the evaluated timing, subtypes of childhood adversities were closely correlated with the epigenetic profiles.

All in all, with respect to the clinical investigation of the ELS-induced epigenetic alterations, the increased DNAm of the NR3C1 promoters, allele-specific FKBP5 DNA demethylation, the hypermethylation of the SLC6A4 promoters, and the increased DNAm of BNDF were associated with ELS. These epigenetic alterations were assumed to increase the risk for depression, and these transformations lasted long into older adulthood or even impacted the offspring (McGowan et al., 2009; Kang et al., 2013; Ouellet-Morin et al., 2013; Wankerl et al., 2014; Montirosso et al., 2016; Tyrka et al., 2016; Fumagalli et al., 2018; Palma-Gudiel et al., 2018; Peng et al., 2018; Roberts et al., 2018; Fiacco et al., 2019; Wikenius et al., 2019). However, regarding individuals who suffered ELS but remained healthy across adulthood, ELS may bring about different or even opposite epigenetic changes. Another possibility is genetic preconditions that may depict the epigenetic profiles resistant or vulnerable to ELS. Therefore, we summarized the research into ELS in Table 1. We emphasized the demographic features and measured methods of epigenetic profiles. Even in studies that supported that ELS was associated with the epigenetic alterations of the disturbed HPA axis, serotonin, and neurogenesis, debates remain as to whether epigenetic changes resulted from ELS or whether those changes were significant enough to promote the risk for depression (Wankerl et al., 2014; Alexander et al., 2018; Fiacco et al., 2019).

**TABLE 1:** Early life stress modulates epigenetic profiles in human.

Participants	Gender	Types of ELS	Measured methods	Epigenetic outcome	Phenotypic bridge	References
Healthy adults	62% F	PA, SA, EA, PN, EN	Pyrosequencing	Glucocorticoid receptor promoter DNAm in leukocyte↓	Healthy adults exposed to ELS differ in epigenetic profile to lifetime depressed patients	Tyrka et al., 2016
Healthy young adults	50% F	PA, SA, EA, PN, EN	Pyrosequencing	1_*F*_ NR3C1 promoter DNAm↑	No direct association between ELS and NR3C1-1_*F*_ DNAM	Alexander et al., 2018
Infants at 2 months of age	M + F	Prenatal depressive symptoms	Pyrosequencing	1_*F*_ NR3C1 promoter DNAm↑ in male infants; IV BDNF promoter DNAm↓ in both gender	No association between maternal cortisol and infant DNA methylation	Braithwaite et al., 2015
Preschool children at the age of 3–5 years	61%F	PA, SA, EA, PN, EN	Pyrosequencing	1_*D*_ and 1_*F*_ NR3C1 promoter DNAm↑	N/A	Tyrka et al., 2015
MDD adults	62%F	Childhood maltreatment (mixed types)	Pyrosequencing	Childhood maltreatment associated with glucocorticoid receptor promoter DNAm in peripheral blood↑	Childhood maltreatment other than depression correlated with NR3C1 expression	Bustamante et al., 2016
Suicide victims	M	Childhood abuse/neglect	Sodium bisulfite mapping	1_*F*_ NR3C1 promoter DNAm↑	NR3C1 expression in hippocampus↓	McGowan et al., 2009
Suicide victims	M	Severe childhood abuse	Immunoprecipitation + microarray	248 hypermethylated and 114 hypomethylated	N/A	Labonté et al., 2012
British adults	M	PA, EA, SA	Methylated DNA immunoprecipitation + pyrosequencing	311 hypermethylated and 686 hypomethylated	N/A	Suderman et al., 2014
Young adults	53% F	PA, SA, EA, PN, EN	Microarray	Stress-related genes differently methylated	Possible increased risk for psychiatric and physical disorders	Cecil et al., 2016
Healthy adults	M	Physical aggression	Pyrosequencing	SLC6A4 promoter DNAm↑	5-HT synthesis↓	Wang et al., 2012
MDD patients	81% F	Parental loss, financial hardship, physical and sexual abuse	Pyrosequencing	SLC6A4 promoter DNAm↑	Worse depressive symptoms	Kang et al., 2013
Very preterm and full-term infants at the age of 3 months	44% F	Socio-emotional stress	Pyrosequencing	SLC6A4 promoter DNAm↑ in very preterm infants	Worse negative emotion	Montirosso et al., 2016
Monozygotic twins	Same-sex within twins	Bullying victimization	Amplified site-specific and evaluated via Sequenom EpiTYPER system	SERT promoter DNAm↑	Blunted cortisol responses to stress	Ouellet-Morin et al., 2013
Healthy young adults	50% F	Maternal prenatal stress/child maltreatment	Pyrosequencing	No significant DNAm alterations associated with ELS	Lower SERT mRNA levels	Wankerl et al., 2014
African American young adults	M	PA, SA, EA, PN, EN	Pyrosequencing	IL6 promoter DNAm↓	Pro-inflammatory response to stress	Janusek et al., 2017
MDD patients	72% F	PA, SA, EA, PN, EN	Microarray	BDNF promoter DNAm↑	BDNF polymorphisms collaborated with ELS	Ferrer et al., 2019

*MDD, major depression disorder; F, female; M, male; EA, emotion abuse; SA, sexual abuse; PA, physical abuse; EN, emotion neglect; PN, physical neglect; DNAm, DNA methylation; NR3C1, neuron-specific glucocorticoid receptor; SLC6A4, serotonin transporter; BDNF, brain-derived neurotrophic factor.*

## Epigenetic Studies Focused on Animals

In addition to clinical research, preclinical studies were also conducted in pursuing the role of ELS-mediated epigenetic profiles in depression. As for the preclinical investigations, rodents were the most used materials. Of behaviors of ELS, maternal deprivation, improper maternal care, and other maltreatments were found to affect the epigenetic profiles of the animals. With regard to the animal models who developed depression, ELS enhanced the effect of adversities and made the models susceptible to depressive symptoms (Zhang et al., 2013, 2015). It was reported that chronic and unpredictable maternal separation (MS) altered the status of DNAm in the promoter of several candidate genes in the germline of the separated males, and the epigenetic characteristics possibly transmitted across generations (Franklin et al., 2010).

Similarly, the epigenetic modifications that occurred in animals were associated with the regulation of the HPA axis, monoamine, and neurogenesis. Firstly, the promoters of glucocorticoid receptors were differently methylated and these alterations were correlated with the heterogeneous ELS and evaluated timing. It was reported that maternal licking and grooming and arched-back nursing negatively correlated with the DNAm level, while being positively associated with the histone acetylation status of the GR promoters or the CpG island shore in the hippocampus (Bockmühl et al., 2015). Both the DNAm and histone acetylation jointly modulated the activity of the glucocorticoid receptor. Moreover, these epigenetic changes persisted into adulthood and can be reversible by a histone deacetylase regulator or methyl supplementation. Specifically, central infusion of a histone deacetylase inhibitor or methyl supplementation removed the difference of DNAm, histone methylation, GR expression, and HPA axis to stress between high maternal and low maternal care (Weaver et al., 2004, 2005). However, the epigenetic alterations may be sex-specific and strain-dependent, because Kundakovic et al. (2013) showed that maternal separation increased hippocampal *Nr3c1* DNAm in C57BL/6J males only, other than in Balb/cJ mouse strains. Furthermore, in C57BL/6J females, decreased hippocampal *BDNF* expression bonded to MS, while MS-induced the increased hippocampal BDNF level in male and female Balb/cJ offspring. Therefore, in confronting ELS, different animal-strains and responded elements showed discrepancies (Kundakovic et al., 2013). It is worth mentioning that the epigenetic changes of inconsistent strains that responded to ELS was also illustrated in a study investigating histone modification. In the model of MS, mice obtained a decreased expression of mRNA encoding the histone deacetylases (HDACs) 1, 3, 7, 8, and 10 in the forebrain neocortex which was followed by an increased expression of acetylated histone H4 proteins. This epigenetic modification was only observed in the Balb/c strain other than the more resilient C57Bl/6 strain. However, the upregulation of the histone deacetylase (HDAC) and histone hypoacetylation level was in an ELS model of MS tested on Sprague–Dawley rats in the ventral tegmental area. While an HDAC inhibitor was able to reverse the histone hypoacetylation and normalize the BDNF level (Shepard et al., 2018). The histone modifications likely occurred as compensation because the reversal of the histone modifications worsened the abnormal emotional symptoms resulting from ELS and interacted with antidepressants (Levine et al., 2012). Back to the conversation of the HPA axis, the corticotrophin-releasing hormone (CRH) is the upper regulator for the cortisol stress response. One study showed that MS was associated with the decreased methylated *crh* promoter and enhanced *crh* transcriptional responses to stress in adulthood (Chen et al., 2012). Moreover, increased histone H3 acetylation was also observed in MS rats, and an enriched environment reversed the epigenetic alterations and alleviated the upgraded CRH level and some phenotypes (Wang et al., 2014). Additionally, arginine vasopressin (AVP), expressed in the hypothalamic paraventricular nucleus, may coordinate with CRH in modulating the release of corticosteroids and adjusting the HPA axis in stress coping. Murgatroyd et al. (2009) showed that ELS led to hypomethylation of a key regulatory region of the Avp gene and was accompanied with consistent Avp expression in the hypothalamus. Moreover, proopiomelanocortin (Pomc), encoding POMC protein which serves as a prohormone for ACTH, was upregulated because of decreased DNAm of the critical region of the Pomc gene. The epigenetic alteration occurred soon after MS and persisted for a long time (Wu et al., 2014). However, another study assumed that the turbulent function of the HPA axis was not attributed to the DNAm status of the exon 1_7_ GR promoter region when the epigenetic profile was measured 7 days after maternal separation in Sprague Dawley rat pups (Daniels et al., 2009). This difference may result from strains and timeline discrepancy. Kosten and Nielsen (2014) found that litter and sex influenced the value of maternal pup licking and licking difference attributed to the discrepancy of DNAm of Nr3c1 exon 1_7_ promoters in the hippocampus and cerebellum in 35-day postnatal Sprague-Dawley rats. Therefore, though a few inconsistent outcomes were possibly derived from heterogeneous ELS model and evaluated timing, ELS was assumed to disturb the normal function of HPA axis via increased DNAm or inhibiting the transcription of GRs (Weaver et al., 2004; Daniels et al., 2009; Kosten and Nielsen, 2014). Furthermore, CRH, AVP, and POMC may collaborate to regulate the release of glucocorticoids (Murgatroyd et al., 2009; Chen et al., 2012; Wang et al., 2014; Wu et al., 2014).

In addition to the theory that ELS-induced epigenetic alterations enhanced the risk for mental sickness, Kinnally et al. (2011) proposed that an increased 5-methylcytosine (5mC) level contributed to the effect of ELS. In this female bonnet macaque study, DNAm did not differ based on early life stress. Although, increased 5-HTT and whole-genome 5mC levels reacted sharply to the ELS (Kinnally et al., 2011). Basically, 5-Hydroxymethylcytosine (5hmC) regulates DNA demethylation, while 5mC mediates DNAm. Adult female mice exposed to ELS and who had anxiety-like behaviors develop found disruptions of hypothalamic 5hmC and possibly an expression of the stress-related genes (Papale et al., 2017). Furthermore, male rats exposed to caregiver maltreatment had higher 5mC levels in the hippocampus and had lower 5hmC levels in the amygdala (Doherty et al., 2016). Moreover, early-life stressful social experiences elevated the level of DNA methyltransferases (Dnmt3a), ten-eleven translocation (Tet3), methyl-CpG-binding protein-2 (MeCP2), and repressor element-1 silencing transcription factor (REST) in the amygdala of adolescents and adults. The alterations of these DNAm regulators were assumed to modulate the level of DNAm and collaborated with the histone modifications that regulate phenotypes in response to ELS and current stressful events (Karen and Rajan, 2019). Therefore, ELS may alter epigenetic profiles linked to gain risk for depression via gene manipulation or global regulators variation.

As for the molecules involved in depression, BDNF is the most well-known. Roth et al. demonstrated that rats exposed to predominately abusive behaviors produced persistent DNAm of *BDNF* in the adult prefrontal cortex and the epigenetic changes transmitted into the next generation (Roth et al., 2009). Similarly, the altered profile of DNAm induced by MS or other adversities was observed in the germline of C57Bl6/J males and Balb/c mice and can be transmitted through generations (Franklin et al., 2010; Kundakovic et al., 2013, 2015; Park et al., 2018; Shepard et al., 2018). Moreover, ELS contributed to decreased histone H3 acetylation levels binding to the BDNF exon I promoter and increased DNMT1 and DNMT3a mRNA levels in the hippocampus (Park et al., 2018). Additionally, maternal deprivation and current stress induced different contents of depressive symptoms in Sprague-Dawley rats. However, it was maternal deprivation but not current stress rats that contributed to downregulated hippocampal BDNF and higher miR-16 expression (Bai et al., 2012). Moreover, female rats exposed to caregiver maltreatment had greater BDNF DNAm in the amygdala and hippocampus (Doherty et al., 2016). With regard to the histone modifications, Seo and colleagues proposed that ELS built the background for the sensitivity of stress via decreased levels of acetylated histone H3 and H4 at BDNF promoter IV and restraint stress enhanced the epigenetic changes and thereafter deteriorated the depressive phenotypes (Seo et al., 2016). Therefore, types of ELS attributed to the epigenetic inhibition of BDNF and decreased the activity of BDNF in modulating neuroplasticity and neurogenesis.

In addition to the neuroplasticity specifically involved in the derived neurotrophic factor, glutamate receptor, and histone modulated regulators were also investigated. The type I metabotropic glutamate receptor (mGluR1) regulated the synaptic plasticity and *Grm1* encodes this protein. Bagot et al. (2012) found that higher pup licking/grooming rats were associated with decreased DNAm and increased levels of histone 3 lysine 9 acetylation and histone 3 lysine 4 trimethylation of *Grm1* in the hippocampus. The histone modification was negatively associated with the DNAm and positively correlated with *Grm1* transcription and translation. Therefore, good maternal care ensured well-operated receptor function (Bagot et al., 2012). Besides, Wang et al. demonstrated that male Sprague-Dawley rats who suffered early-life social isolation had increased levels of neuronal H3K9me2 (a repressive marker of transcription) in the hippocampus, accompanied by decreased expression of hippocampal N-methyl-D-aspartate (NMDA) receptor subunits, and the AMPA receptor subunits, GluR1 and GluR2. Thus, the epigenetic changes disturbed or deteriorated the neural plasticity, while antidepressants that reversed the epigenetic changes restored the neuroplasticity (Wang et al., 2017). As regards to the histone modulators, dynamic changes differed. Specifically, ELS decreased the level of H3K14 acetylation (ac) and H3K9ac in adolescents and then increased it in adults. While H3K4 methylation (me2/me3) levels were elevated in adolescents and adults, and H3K9me2/me3 levels increased in adults (Karen and Rajan, 2019). Moreover, histone modifications also modulated the pro-inflammatory characteristics in rats exposed to the ELS. It was assumed that chronic unpredictable mild stress during the adolescence period combined with maternal separation induced depressive-like behaviors, burst cytokines, and increased Jmjd3 (a histone H3 lysine 27 (H3K27) demethylase) and decreased H3K27me3 expression in the prefrontal cortex and hippocampus of both adolescent and adult rats (Wang et al., 2018, 2020). Therefore, compared with the studies of the site-specific profile of DNAm, DNAm, and histone regulators seemed to have a more profound effect. Further studies should focus on finding more targets of these modulated regulators.

With respect to the miRNAs, epigenome or regional miRNAs have been investigated. MS or chronic unpredictable mild stress increased the level of miR-16, miR-504, miR-326, miR124a, and decreased miR-9 and miR-135a in respective regions (Bai et al., 2012; Zhang et al., 2013, 2015; Bahi, 2016; Liu et al., 2017; Xu et al., 2017). Of these microRNAs, miR-16, and miR124a correlated negatively with BDNF expression in the rats’ hippocampus which ELS exposed (Bai et al., 2012; Bahi, 2016), and miR-504 was negatively related with lower dopamine receptor D1 (DRD1) and D2 (DRD2) expression in the nucleus accumbens of the rats (Zhang et al., 2013). While in another study, maternal deprivation-induced DRD2 mRNA expression was accompanied by decreased miR-9 in the striatum (Zhang et al., 2015). However, although substantial alterations of miRNAs were observed in ELS models, the precise mechanisms of how and what these miRNAs transcriptions regulated are unknown. Consequently, the underlying relationship between miRNAs and depressive phenotypes needs to be clarified and how miRNA interacted with DNAm and histone modification requires more exploration.

In conclusion, according to the studies that focused on the non-human animals, ELS was associated with an increased DNAm of the glucocorticoid receptor and BDNF (Weaver et al., 2004; Kosten and Nielsen, 2014; Doherty et al., 2016; Park et al., 2018). While genes contributing to glucocorticoid release were upregulated. Additionally, alterations of histone modifications binding to specific regions or genome-wide and miRNAs also led to decreased GR and BDNF expression, while permitted the CRH, AVP, and POMC expression (Murgatroyd et al., 2009; Chen et al., 2012; Wang et al., 2014; Wu et al., 2014). The epigenetic modifications reacting to ELS were listed in Table 2. These changes were assumed to result in the aberrant HPA axis and disrupt neurogenesis and neuroplasticity. Moreover, compared with the epigenetic investigations focused on the human subjects, the non-human studies showed more consistent outcomes when strains and ELS interventions remained the same (Kundakovic et al., 2013; Kosten and Nielsen, 2014).

**TABLE 2:** Early life stress modulates epigenetic profiles in non-human animals.

Animal strains	Types of ELS	Measured methods	Epigenetic outcome	Phenotypic bridge	References
Long-Evans hooded rats	Decreased maternal care	Sodium bisulfite mapping + Chromatin immunoprecipitation	GR promoter DNAm↑ and histone acetylation↓	Impaired HPA axis to stress and can be reversed by the environment or medication	Weaver et al., 2004, 2005
Sprague Dawley rats	MS/MD	Site-specific accumulation and sequencing	No GR promoter DNAm occurred at 21 days postnatal	Dysfunctional HPA axis not associated with GR epigenetic profile	Daniels et al., 2009
**Sprague Dawley rats**	MS/MD	Pyrosequencing	CpGs of CRH promoter DNAm↑	HPA axis hypersensitivity	Chen et al., 2012
Sprague Dawley rats	MS/MD	Immunoprecipitation	Histone H3 acetylation↑ and cytosine methylation↓ in Crh promoter region	Hippocampal synaptic dysfunction and memory defects	Wang et al., 2014
C57Bl6/J **mice**	MS/MD	Pyrosequencing	Altered germline DNAm profile in males only	Male offspring easily obtained epigenetic profiles	Franklin et al., 2010
Female bonnet macaques	Programmed adversities	Pyrosequencing + Whole-genome methylation ELISA	Whole-genome 5mC↑	Aberrant stress reactivity	Kinnally et al., 2011
C57BL/6J and Balb/cJ **mice**	MS/MD	Pyrosequencing	BDNF DNAm↑ in Balb/c mice and Nr3c1 DNAm↑ in C57BL/6J males	The discrepancy between the epigenetic profile and gene expression. Sex and strains differed epigenetically	Kundakovic et al., 2013
**Sprague Dawley rats**	MS/MD	Immunoprecipitation	Histone H3 acetylation↓ and DNMT1 and DNMT3a↑	Decreased BDNF protein	Park et al., 2018
Wistar rats	Early-life stressful social experience	Quantitative real-time PCR + Western blot	Dnmt3a↑, H3K14ac↑, and H3K9ac↓	Decreased BDNF protein	Karen and Rajan, 2019
Sprague Dawley rats	MS/MD	Quantitative real-time PCR + Chromatin immunoprecipitation	Acetylated histone H3 and H4 at BDNF promoter IV↓	Decreased BDNF protein	Seo et al., 2016
**Balb/cJ and C57Bl/6J mice**	MS/MD	Real-time RT-PCR + Western blot	Histone deacetylases↓ and acetylated histone H4 proteins↑ in Balb/c mice but not C57Bl/6mice	Medications helped to modulate epigenetic profiles/alleviated depressive symptoms	Levine et al., 2012
Sprague Dawley **rats**	Decreased maternal care	Pyrosequencing	GR promoter DNAm↑ in hippocampus and cerebellum	Litter and sex differed in epigenetic profiles	Kosten and Nielsen, 2014
Long-Evans outbred rats	Caregiver maltreatment	Global DNA and Locus-specific detection	5-mC↑ in males’ hippocampus, 5-hmC↓ in males’ amygdala, BDNF DNA↑ in males’ and females’ amygdala and hippocampus	Sex-specific and region-specific DNAm profiles in the whole genome	(Doherty et al., 2016)
C57BL/6J mice	Early-life environmental stress	Genome-wide sequencing	Altered 5hmC level	Depressive symptoms	Papale et al., 2017
C57BL/6 mice	MS/MD	Immunoprecipitation + ChIP-seq	Altered genomic landscape of H3K4me3	Reduced locomotor activity and reduced exploratory activity	Ershov et al., 2018
Sprague Dawley rats	MS/MD	Real-time reverse transcription PCR + Western blot	miRNA-504↑	Decreased dopamine receptor D2 expression	Zhang et al., 2013
Sprague Dawley rats	MS/MD	Real-Time reverse transcription quantitative PCR + Western blot	miRNA-9↓ and miRNA-326↑ in the striatum	Increased dopamine receptor D2 expression	Zhang et al., 2015

*MS, maternal separation; MD, maternal deprivation; GR, glucocorticoid receptor; 5-mC, 5-methylcytosine; 5-hmC, 5-hydroxymethylcytosine; DNMT, DNA methyltransferase; BDNF, brain-derived neurotrophic factor.*

## Conclusion and Discussion

ELS is prevalent among humans, and the relationship among ELS, the dynamic epigenetic alterations, and depression have been investigated in recent years. Epidemiological studies encouraged that ELS could possibly increase the risk of depression and enhance the adverse effect of later life stress. However, different types of early adversities and sex had considerable effects on the consequences (Kim et al., 2013; Culpin et al., 2015; St Clair et al., 2015; Bjorkenstam et al., 2017; Dahl et al., 2017; Frodl et al., 2017; Opel et al., 2019). The epigenetic alterations may help to explain how ELS modulated the epigenetic reprogramming and what the effect of these alterations was. Therefore, we cited the evidence that displayed the relationship, and most studies agreed that epigenetic changes were observed in humans and animal models following ELS (Weaver et al., 2004; McGowan et al., 2009; Chen et al., 2012; Kang et al., 2013; Ouellet-Morin et al., 2013; Bockmühl et al., 2015; Bustamante et al., 2016; Montirosso et al., 2016; Tyrka et al., 2016; Palma-Gudiel et al., 2018; Peng et al., 2018). Compared with individuals free of ELS, early life adversities were associated with substantial epigenetic alterations. The epigenetic drifts modulated the function of the HPA axis, monoamine, and neurogenesis and neuroplasticity (Weaver et al., 2004; McGowan et al., 2009; Perroud et al., 2011; Chen et al., 2012; Wang et al., 2014; Bockmühl et al., 2015; Tyrka et al., 2016; Palma-Gudiel et al., 2018; Peng et al., 2018). As for the studies, they mostly supported that ELS increased the risk of depression and that epigenetic reprogramming contributed to the dysfunctional response to stress. Specifically, at least altered DNAm and histone activity helped to deactivate GR and destroy the negative feedback regulation of the HPA axis. The abnormal HPA axis function decreased the threshold for developing depression.

In addition to the regulation of the HPA axis, dysregulated monoamine in the brain or peripheral blood resulting from epigenetic adjustments were also observed. The alternative epigenetic changes of 5-HT were illustrated. Increased DNAm or histone modifications of 5-HTT related genes were associated with ELS (Kinnally et al., 2011; Kang et al., 2013; Melas et al., 2013; Wankerl et al., 2014; Montirosso et al., 2016; Fumagalli et al., 2018). While exhausted 5-HT in the synapse and the blunt cortisol response enlarged the adverse event in later life (Ouellet-Morin et al., 2013). Therefore, it was likely epigenetic regulations that modulated the background for depression development and deterioration in the context of ELS.

Furthermore, molecules involved in the neurogenesis and neuroplasticity were also reshaped corresponding to the ELS. According to most of the evidence, increased DNAm and altered histone modifications of the genes promoted neurogenesis and neuroplasticity which was also observed in individuals or animals with ELS (Roth et al., 2009; Bai et al., 2012; Doherty et al., 2016; Seo et al., 2016; Ferrer et al., 2019). The imbalanced epigenetic modulations bestowed susceptibility to depressive symptoms.

The profile of miRNAs also changed when confronted with ELS and played a role in promoting depression. Depression and ELS bilaterally contributed to the dynamic turbulence of miR-16, miR-504, miR-326, miR124a, miR-9, and miR-135a. However, though part of the network of miRNAs was illustrated, how these collaborated remains unknown. Furthermore, what the targets are of these miRNAs stayed largely undisclosed (Bai et al., 2012; Zhang et al., 2013, 2015; Bahi, 2016; Liu et al., 2017; Xu et al., 2017). Of note, broad research should analyze the network for these miRNAs.

As regards the epigenome-wide study, a number of epigenetic adaptations were discovered. Meanwhile, because of the large amounts of influenced genes discovered and dynamic epigenetic regulations across life, an assortment of biological activities were involved (Franklin et al., 2010; Kinnally et al., 2011; Doherty et al., 2016; Papale et al., 2017; Wikenius et al., 2019). However, it is difficult to qualitatively and quantitatively measure the role of these changes induced by ELS and how much they accounted for the onset of depression.

Apart from the research that supported the role of early life stress on the epigenetic profiles in depression, few researchers assumed that no significant evidence of epigenetic changes following ELS increased the risk for depression or that ELS was not responsible for depression formation based on epigenetic proof (Daniels et al., 2009; Kundakovic et al., 2013; Bustamante et al., 2016; Alexander et al., 2018; Fiacco et al., 2019). One theory of depression requires two-hit or multiple-hit stressful events. The development of depression requires early adversities and the following burden strengthens ill-programmed epigenetic profiles. In the individuals who were exposed to ELS but remained healthy in adults, the epigenetic profiles differed compared with the individuals who later developed depression. Furthermore, genetic background and risk of polymorphisms of genes were more likely to obtain epigenetic reprogramming tilted toward depression (Peña et al., 2013; McCoy et al., 2016; Alexander et al., 2018; Ferrer et al., 2019). Additionally, the contents and severity of ELS was associated with different epigenetic profiles. In human, physical abuse, sexual abuse, emotional abuse, and physical & emotional neglects were the most studied (Kim et al., 2013; Culpin et al., 2015; St Clair et al., 2015; Airagnes et al., 2016; Opel et al., 2019). While in non-human research, maternal deprivation/separation and prenatal stress were greatly explored. Besides, especially for the human beings, consistent stress continuously reprogramed the epigenetic profiles (Weaver et al., 2004; Murgatroyd et al., 2009; Roth et al., 2009; Zhang et al., 2013; Seo et al., 2016; Reus et al., 2017; Xu et al., 2017). Additionally, ELS was mostly measured retrospectively in humans and commonly individuals suffered from more than one adversity (St Clair et al., 2015; Williams et al., 2016; Bjorkenstam et al., 2017; Opel et al., 2019). This situation likely contributed to heterogeneous outcomes when epigenetic characteristics were measured. Furthermore, with regard to the studies of non-human animals, though the models of ELS were well-controlled, the strain and measured timing possibly led to incongruous results (Weaver et al., 2004; Murgatroyd et al., 2009; Chen et al., 2012; Levine et al., 2012; Seo et al., 2016). Moreover, sex differences influenced the development of depression due to ELS in both human and non-human animals (Kundakovic et al., 2013; Melas et al., 2013; Kosten and Nielsen, 2014; St Clair et al., 2015; Frodl et al., 2017). Although compared with humans, the epigenetic profiles correlated with ELS of non-human animals were relatively consistent when sex, strain, and measured methods remained the same (Weaver et al., 2004, 2005; Murgatroyd et al., 2009; Wang et al., 2014).

**FIGURE 1:**
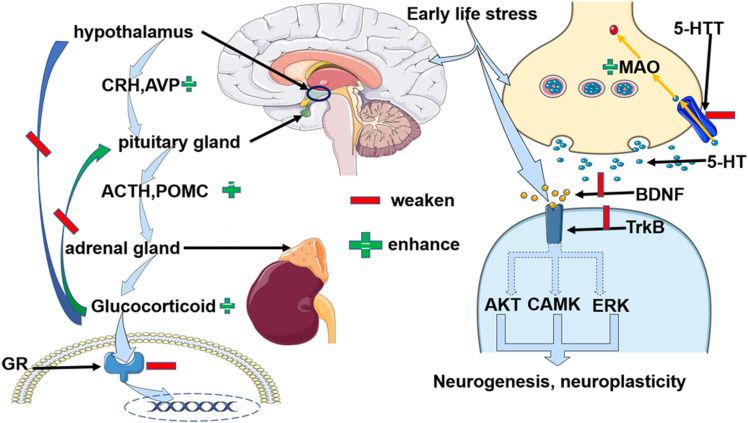
Early life stress-induced alterations of three depressive-related paths, the HPA axis, 5-HT, and BNDF, respectively due to epigenetic modification. 1) Early life stress impaired the GRs and paralleled with the dysfunctional negative feedback loop of the HPA axis. Specifically, the enhanced expression of CRH and ACTH, and abundant glucocorticoid was observed. Moreover, AVP, POMC, and FKBP5 KITLG collaborated to regulate the function of HPA axis.2) Early life stress primarily weakened the activity of 5-HTT but enhanced MAO activity. Therefore, early life stress suppressed the recycling of 5-HT and facilitated its degradation. 3) Early life stress inhibited the BDNF and TrkB and indirectly impaired the neurogenesis and neuroplasticity via AKT, CAMK, and ERK pathways. Abbreviation: CRH: corticotrophin-releasing hormone; AVP: arginine vasopressin, ACTH: adrenocorticotropic hormone; POMC: proopiomelanocortin, GR: glucocorticoid receptor; 5-HT: 5-hydroxytryptamine; 5-HTT: serotonin transporter; BDNF: brain-derived neurotrophic factor; TrkB: tropomyosin receptor kinase B; CAMK: calmodulin-dependent kinase, FKBP5: FK506 binding protein 5, KITLG: Kit ligand gene.

In conclusion, ELS might increase the risk of depression by building a susceptible background for the depression (Kim et al., 2013; Bjorkenstam et al., 2017; Dahl et al., 2017; Opel et al., 2019). Epigenetic alterations may add fuel to the fire in the development of depression when individuals possess a history of early life stress and a risk genetic property. We concluded that the possible epigenetic outcomes of ELS induced changes that shared common biological changes with depression as in Figure 1. Due to the fact that the epigenetic changes suppressed the expression of GR, and elevated CRH, AVP, and ACTH expression, the HPA axis was dysfunctional. Specifically, negative feedback was destroyed and continuously higher glucocorticoids circulated within the body. An excessive amount of glucocorticoids affected inflammatory and neuroplastic activities. On the other hand, ELS decreased the expression of 5-HTT and inhibited the transportation of 5-HT to the presynaptic neurons. In the meantime, enhanced monoamine oxidase (MAO) degraded the 5-HT. Moreover, in the subsynaptic membrane, decreased BDNF expression selectively resulted in knockdown of the TrkB receptor which led to the adverse effect on the neurogenesis and neuroplasticity (Weaver et al., 2004, 2005; McGowan et al., 2009; Chen et al., 2012; Kang et al., 2013; Ouellet-Morin et al., 2013; Tyrka et al., 2016; Peng et al., 2018; Prowse et al., 2020). More research needs to be done to investigate the direct evidence for the role of early life stress-induced epigenetic changes when contributing to the vulnerability of depression because of the uncertainty proposed by finite evidence. Furthermore, scientists should move on to explore the dynamic epigenetic adaptations when individuals confronted ELS and later depression. It is possible that a group of people obtained ELS when the epigenetic program was active, but remained healthy in later life, epigenetic alterations could be an adaptive evolution. Therefore, exploring the regulators to induce the epigenetic changes to accommodate stress events will be promising.

## Author Contributions

ML wrote the first draft. XF, WX, WG, BL, RC, and WY made major revisions to the logic of this article. WY provided critical revisions. All authors approved the final version of the manuscript for submission.

## Conflict of Interest

The authors declare that the research was conducted in the absence of any commercial or financial relationships that could be construed as a potential conflict of interest.
